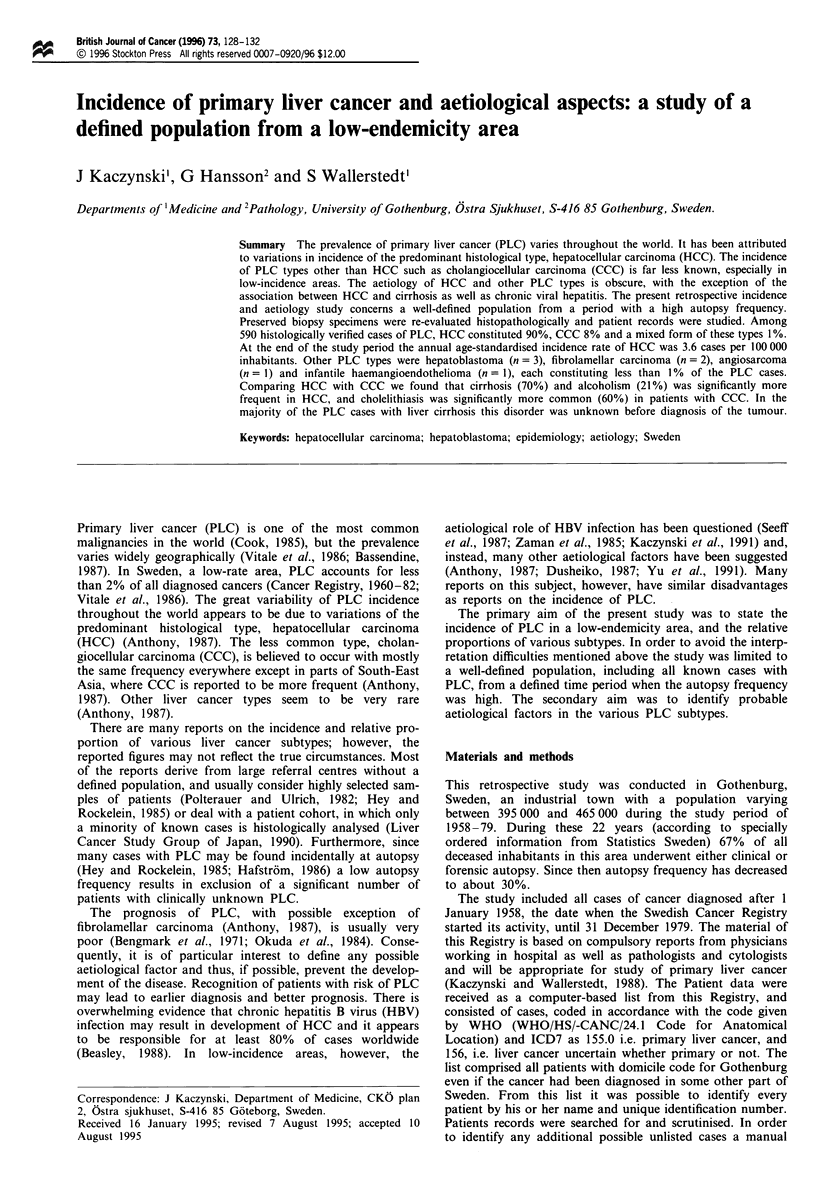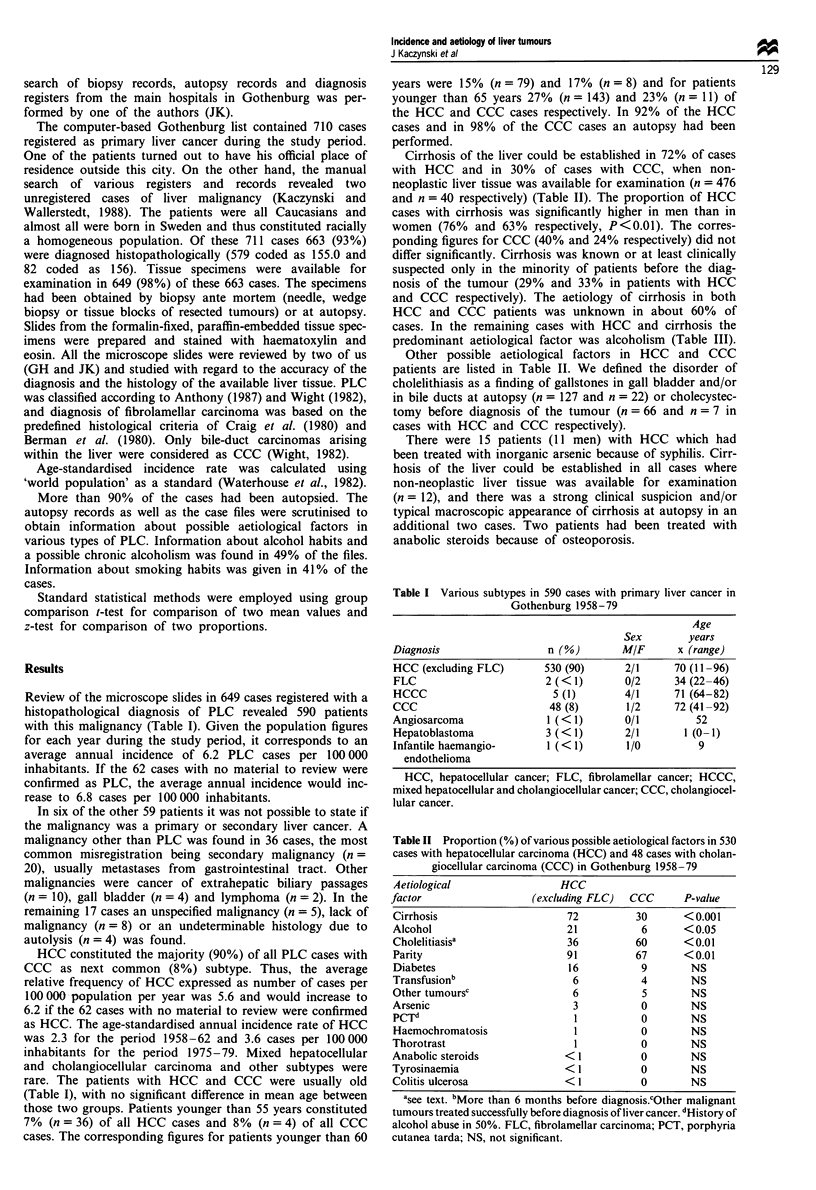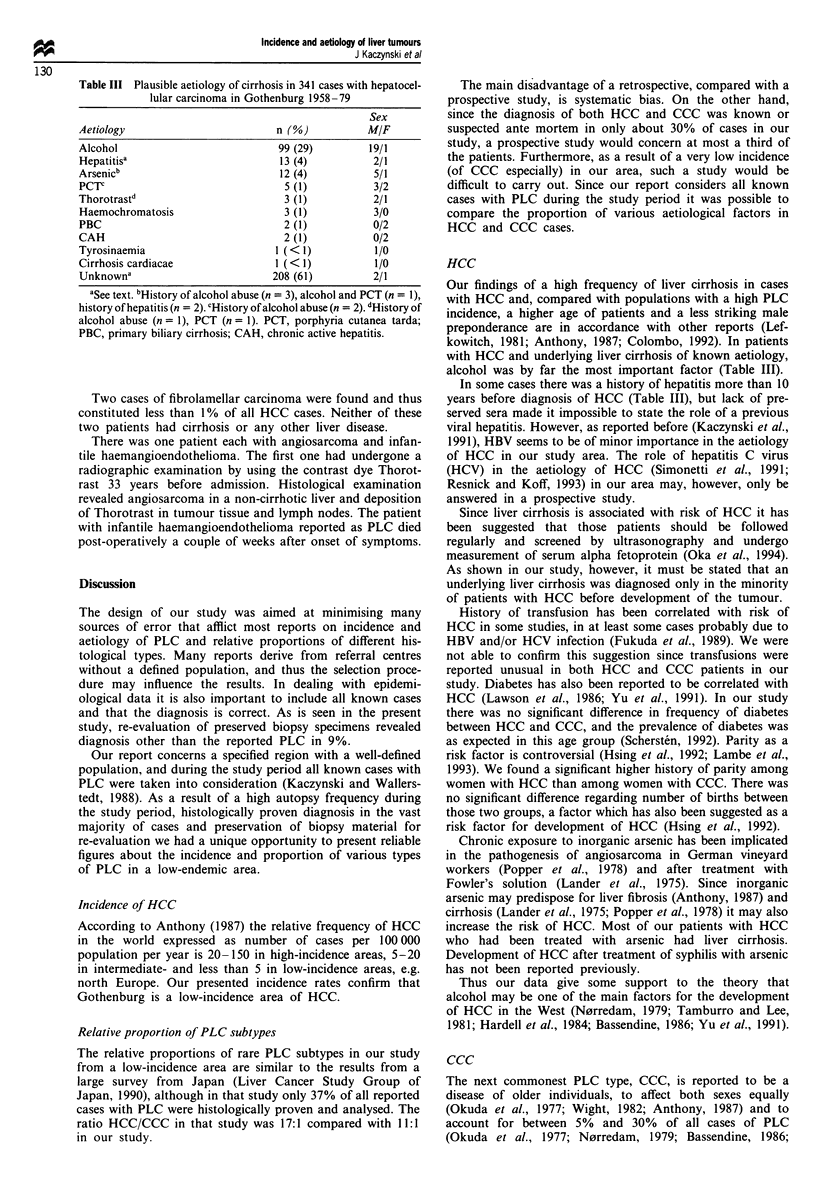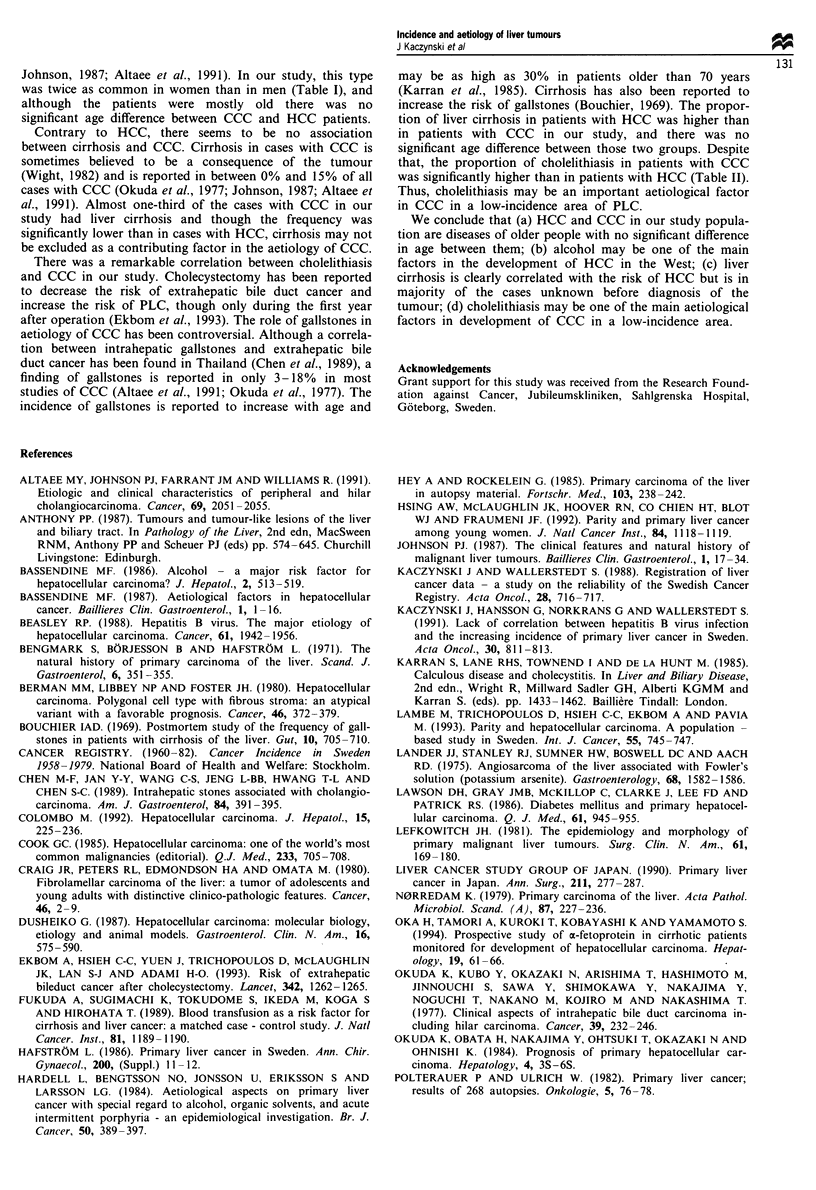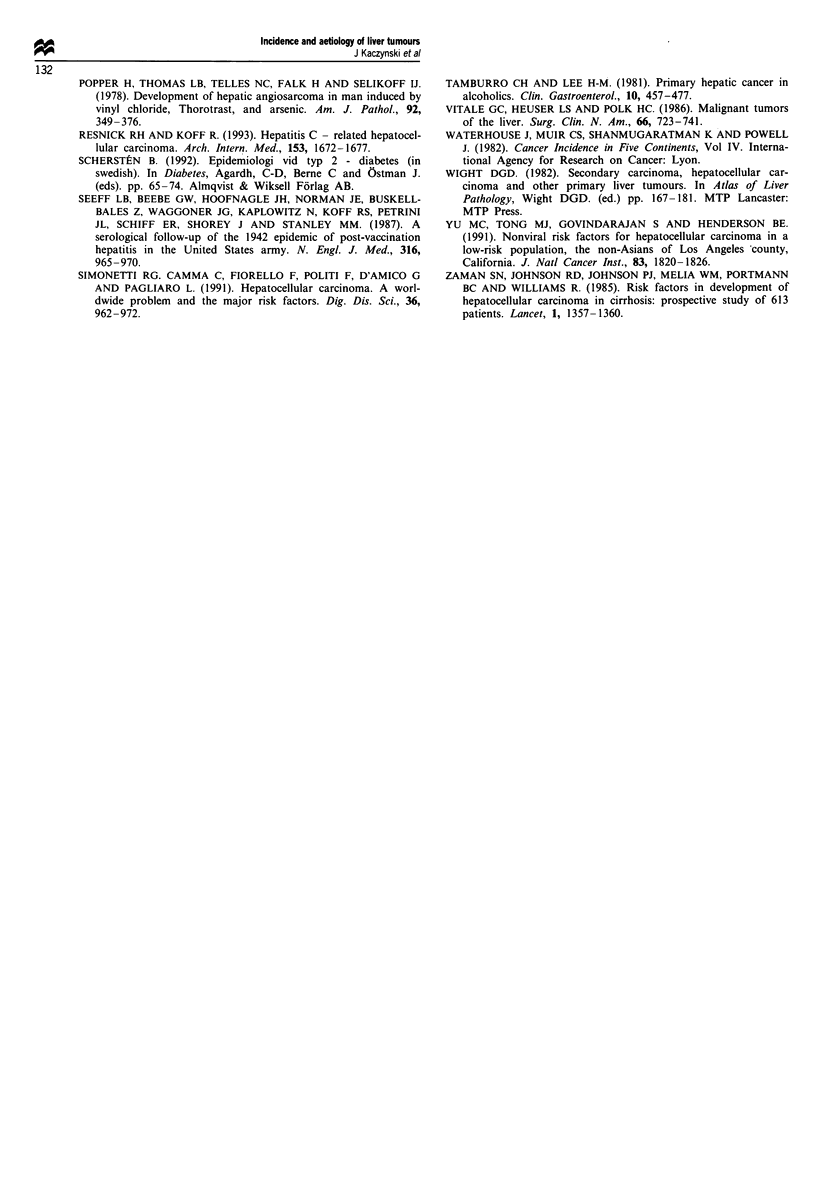# Incidence of primary liver cancer and aetiological aspects: a study of a defined population from a low-endemicity area.

**DOI:** 10.1038/bjc.1996.24

**Published:** 1996-01

**Authors:** J. Kaczynski, G. Hansson, S. Wallerstedt

**Affiliations:** Department of Medicine, University of Gothenburg, Ostra Sjukhuset, Sweden.

## Abstract

The prevalence of primary liver cancer (PLC) varies throughout the world. It has been attributed to variations in incidence of the predominant histological type, hepatocellular carcinoma (HCC). The incidence of PLC types other than HCC such as cholangiocellular carcinoma (CCC) is far less known, especially in low-incidence areas. The aetiology of HCC and other PLC types is obscure, with the exception of the association between HCC and cirrhosis as well as chronic viral hepatitis. The present retrospective incidence and aetiology study concerns a well-defined population from a period with a high autopsy frequency. Preserved biopsy specimens were re-evaluated histopathologically and patient records were studied. Among 590 histologically verified cases of PLC, HCC constituted 90%, CCC 8% and a mixed form of these types 1%. At the end of the study period the annual age-standardised incidence rate of HCC was 3.6 cases per 100,000 inhabitants. Other PLC types were hepatoblastoma (n = 3), fibrolamellar carcinoma (n = 2), angiosarcoma (n = 1) and infantile haemangioendothelioma (n = 1), each constituting less than 1% of the PLC cases. Comparing HCC with CCC we found that cirrhosis (70%) and alcoholism (21%) was significantly more frequent in HCC, and cholelithiasis was significantly more common (60%) in patients with CCC. In the majority of the PLC cases with liver cirrhosis this disorder was unknown before diagnosis of the tumour.


British Journal of Cancer (1996) 73, 128-132
© 1996 Stockton Press All rights reserved 0007-0920/96 $12.00

### Incidence of primary liver cancer and aetiological aspects: a study of a defined population from a low-endemicity area

J Kaczynski[1], G Hansson[2] and S Wallerstedt[1]

*Departments of [1]Medicine and [2]Pathology, University of Gothenburg, Östra Sjukhuset, S-416 85 Gothenburg, Sweden.*

**Summary** The prevalence of primary liver cancer (PLC) varies throughout the world. It has been attributed to variations in incidence of the predominant histological type, hepatocellular carcinoma (HCC). The incidence of PLC types other than HCC such as cholangiocellular carcinoma (CCC) is far less known, especially in low-incidence areas. The aetiology of HCC and other PLC types is obscure, with the exception of the association between HCC and cirrhosis as well as chronic viral hepatitis. The present retrospective incidence and aetiology study concerns a well-defined population from a period with a high autopsy frequency. Preserved biopsy specimens were re-evaluated histopathologically and patient records were studied. Among 590 histologically verified cases of PLC, HCC constituted 90%, CCC 8% and a mixed form of these types 1%. At the end of the study period the annual age-standardised incidence rate of HCC was 3.6 cases per 100 000 inhabitants. Other PLC types were hepatoblastoma ($n = 3$), fibrolamellar carcinoma ($n = 2$), angiosarcoma ($n = 1$) and infantile haemangioendothelioma ($n = 1$), each constituting less than 1% of the PLC cases. Comparing HCC with CCC we found that cirrhosis (70%) and alcoholism (21%) was significantly more frequent in HCC, and cholelithiasis was significantly more common (60%) in patients with CCC. In the majority of the PLC cases with liver cirrhosis this disorder was unknown before diagnosis of the tumour.

**Keywords:** hepatocellular carcinoma; hepatoblastoma; epidemiology; aetiology; Sweden

Primary liver cancer (PLC) is one of the most common malignancies in the world (Cook, 1985), but the prevalence varies widely geographically (Vitale *et al.*, 1986; Bassendine, 1987). In Sweden, a low-rate area, PLC accounts for less than 2% of all diagnosed cancers (Cancer Registry, 1960-82; Vitale *et al.*, 1986). The great variability of PLC incidence throughout the world appears to be due to variations of the predominant histological type, hepatocellular carcinoma (HCC) (Anthony, 1987). The less common type, cholangiocellular carcinoma (CCC), is believed to occur with mostly the same frequency everywhere except in parts of South-East Asia, where CCC is reported to be more frequent (Anthony, 1987). Other liver cancer types seem to be very rare (Anthony, 1987).

There are many reports on the incidence and relative proportion of various liver cancer subtypes; however, the reported figures may not reflect the true circumstances. Most of the reports derive from large referral centres without a defined population, and usually consider highly selected samples of patients (Polterauer and Ulrich, 1982; Hey and Rockelein, 1985) or deal with a patient cohort, in which only a minority of known cases is histologically analysed (Liver Cancer Study Group of Japan, 1990). Furthermore, since many cases with PLC may be found incidentally at autopsy (Hey and Rockelein, 1985; Hafström, 1986) a low autopsy frequency results in exclusion of a significant number of patients with clinically unknown PLC.

The prognosis of PLC, with possible exception of fibrolamellar carcinoma (Anthony, 1987), is usually very poor (Bengmark *et al.*, 1971; Okuda *et al.*, 1984). Consequently, it is of particular interest to define any possible aetiological factor and thus, if possible, prevent the development of the disease. Recognition of patients with risk of PLC may lead to earlier diagnosis and better prognosis. There is overwhelming evidence that chronic hepatitis B virus (HBV) infection may result in development of HCC and it appears to be responsible for at least 80% of cases worldwide (Beasley, 1988). In low-incidence areas, however, the aetiological role of HBV infection has been questioned (Seeff *et al.*, 1987; Zaman *et al.*, 1985; Kaczynski *et al.*, 1991) and, instead, many other aetiological factors have been suggested (Anthony, 1987; Dusheiko, 1987; Yu *et al.*, 1991). Many reports on this subject, however, have similar disadvantages as reports on the incidence of PLC.

The primary aim of the present study was to state the incidence of PLC in a low-endemicity area, and the relative proportions of various subtypes. In order to avoid the interpretation difficulties mentioned above the study was limited to a well-defined population, including all known cases with PLC, from a defined time period when the autopsy frequency was high. The secondary aim was to identify probable aetiological factors in the various PLC subtypes.

### Materials and methods

This retrospective study was conducted in Gothenburg, Sweden, an industrial town with a population varying between 395 000 and 465 000 during the study period of 1958-79. During these 22 years (according to specially ordered information from Statistics Sweden) 67% of all deceased inhabitants in this area underwent either clinical or forensic autopsy. Since then autopsy frequency has decreased to about 30%.

The study included all cases of cancer diagnosed after 1 January 1958, the date when the Swedish Cancer Registry started its activity, until 31 December 1979. The material of this Registry is based on compulsory reports from physicians working in hospital as well as pathologists and cytologists and will be appropriate for study of primary liver cancer (Kaczynski and Wallerstedt, 1988). The Patient data were received as a computer-based list from this Registry, and consisted of cases, coded in accordance with the code given by WHO (WHO/HS/-CANC/24.1 Code for Anatomical Location) and ICD7 as 155.0 i.e. primary liver cancer, and 156, i.e. liver cancer uncertain whether primary or not. The list comprised all patients with domicile code for Gothenburg even if the cancer had been diagnosed in some other part of Sweden. From this list it was possible to identify every patient by his or her name and unique identification number. Patients records were searched for and scrutinised. In order to identify any additional possible unlisted cases a manual

Correspondence: J Kaczynski, Department of Medicine, CKÖ plan 2, Östra sjukhuset, S-416 85 Göteborg, Sweden.
Received 16 January 1995; revised 7 August 1995; accepted 10 August 1995

search of biopsy records, autopsy records and diagnosis registers from the main hospitals in Gothenburg was performed by one of the authors (JK).

The computer-based Gothenburg list contained 710 cases registered as primary liver cancer during the study period. One of the patients turned out to have his official place of residence outside this city. On the other hand, the manual search of various registers and records revealed two unregistered cases of liver malignancy (Kaczynski and Wallerstedt, 1988). The patients were all Caucasians and almost all were born in Sweden and thus constituted racially a homogeneous population. Of these 711 cases 663 (93%) were diagnosed histopathologically (579 coded as 155.0 and 82 coded as 156). Tissue specimens were available for examination in 649 (98%) of these 663 cases. The specimens had been obtained by biopsy ante mortem (needle, wedge biopsy or tissue blocks of resected tumours) or at autopsy. Slides from the formalin-fixed, paraffin-embedded tissue specimens were prepared and stained with haematoxylin and eosin. All the microscope slides were reviewed by two of us (GH and JK) and studied with regard to the accuracy of the diagnosis and the histology of the available liver tissue. PLC was classified according to Anthony (1987) and Wight (1982), and diagnosis of fibrolamellar carcinoma was based on the predefined histological criteria of Craig *et al.* (1980) and Berman *et al.* (1980). Only bile-duct carcinomas arising within the liver were considered as CCC (Wight, 1982).

Age-standardised incidence rate was calculated using 'world population' as a standard (Waterhouse *et al.*, 1982).

More than 90% of the cases had been autopsied. The autopsy records as well as the case files were scrutinised to obtain information about possible aetiological factors in various types of PLC. Information about alcohol habits and a possible chronic alcoholism was found in 49% of the files. Information about smoking habits was given in 41% of the cases.

Standard statistical methods were employed using group comparison $t$-test for comparison of two mean values and $z$-test for comparison of two proportions.

### Results

Review of the microscope slides in 649 cases registered with a histopathological diagnosis of PLC revealed 590 patients with this malignancy (Table I). Given the population figures for each year during the study period, it corresponds to an average annual incidence of 6.2 PLC cases per 100 000 inhabitants. If the 62 cases with no material to review were confirmed as PLC, the average annual incidence would increase to 6.8 cases per 100 000 inhabitants.

In six of the other 59 patients it was not possible to state if the malignancy was a primary or secondary liver cancer. A malignancy other than PLC was found in 36 cases, the most common misregistration being secondary malignancy ($n$ = 20), usually metastases from gastrointestinal tract. Other malignancies were cancer of extrahepatic biliary passages ($n$ = 10), gall bladder ($n$ = 4) and lymphoma ($n$ = 2). In the remaining 17 cases an unspecified malignancy ($n$ = 5), lack of malignancy ($n$ = 8) or an undeterminable histology due to autolysis ($n$ = 4) was found.

HCC constituted the majority (90%) of all PLC cases with CCC as next common (8%) subtype. Thus, the average relative frequency of HCC expressed as number of cases per 100 000 population per year was 5.6 and would increase to 6.2 if the 62 cases with no material to review were confirmed as HCC. The age-standardised annual incidence rate of HCC was 2.3 for the period 1958–62 and 3.6 cases per 100 000 inhabitants for the period 1975–79. Mixed hepatocellular and cholangiocellular carcinoma and other subtypes were rare. The patients with HCC and CCC were usually old (Table I), with no significant difference in mean age between those two groups. Patients younger than 55 years constituted 7% ($n$ = 36) of all HCC cases and 8% ($n$ = 4) of all CCC cases. The corresponding figures for patients younger than 60 years were 15% ($n$ = 79) and 17% ($n$ = 8) and for patients younger than 65 years 27% ($n$ = 143) and 23% ($n$ = 11) of the HCC and CCC cases respectively. In 92% of the HCC cases and in 98% of the CCC cases an autopsy had been performed.

Cirrhosis of the liver could be established in 72% of cases with HCC and in 30% of cases with CCC, when non-neoplastic liver tissue was available for examination ($n$ = 476 and $n$ = 40 respectively) (Table II). The proportion of HCC cases with cirrhosis was significantly higher in men than in women (76% and 63% respectively, $P<0.01$). The corresponding figures for CCC (40% and 24% respectively) did not differ significantly. Cirrhosis was known or at least clinically suspected only in the minority of patients before the diagnosis of the tumour (29% and 33% in patients with HCC and CCC respectively). The aetiology of cirrhosis in both HCC and CCC patients was unknown in about 60% of cases. In the remaining cases with HCC and cirrhosis the predominant aetiological factor was alcoholism (Table III).

Other possible aetiological factors in HCC and CCC patients are listed in Table II. We defined the disorder of cholelithiasis as a finding of gallstones in gall bladder and/or in bile ducts at autopsy ($n$ = 127 and $n$ = 22) or cholecystectomy before diagnosis of the tumour ($n$ = 66 and $n$ = 7 in cases with HCC and CCC respectively).

There were 15 patients (11 men) with HCC which had been treated with inorganic arsenic because of syphilis. Cirrhosis of the liver could be established in all cases where non-neoplastic liver tissue was available for examination ($n$ = 12), and there was a strong clinical suspicion and/or typical macroscopic appearance of cirrhosis at autopsy in an additional two cases. Two patients had been treated with anabolic steroids because of osteoporosis.

**Table I** Various subtypes in 590 cases with primary liver cancer in Gothenburg 1958–79

| *Diagnosis* | n *(%)* | *Sex M/F* | *Age years* x *(range)* |
|---|---|---|---|
| HCC (excluding FLC) | 530 (90) | 2/1 | 70 (11–96) |
| FLC | 2 (<1) | 0/2 | 34 (22–46) |
| HCCC | 5 (1) | 4/1 | 71 (64–82) |
| CCC | 48 (8) | 1/2 | 72 (41–92) |
| Angiosarcoma | 1 (<1) | 0/1 | 52 |
| Hepatoblastoma | 3 (<1) | 2/1 | 1 (0–1) |
| Infantile haemangio-endothelioma | 1 (<1) | 1/0 | 9 |

HCC, hepatocellular cancer; FLC, fibrolamellar cancer; HCCC, mixed hepatocellular and cholangiocellular cancer; CCC, cholangiocellular cancer.

**Table II** Proportion (%) of various possible aetiological factors in 530 cases with hepatocellular carcinoma (HCC) and 48 cases with cholangiocellular carcinoma (CCC) in Gothenburg 1958–79

| *Aetiological factor* | *HCC (excluding FLC)* | *CCC* | *P-value* |
|---|---|---|---|
| Cirrhosis | 72 | 30 | <0.001 |
| Alcohol | 21 | 6 | <0.05 |
| Cholelitiasis[a] | 36 | 60 | <0.01 |
| Parity | 91 | 67 | <0.01 |
| Diabetes | 16 | 9 | NS |
| Transfusion[b] | 6 | 4 | NS |
| Other tumours[c] | 6 | 5 | NS |
| Arsenic | 3 | 0 | NS |
| PCT[d] | 1 | 0 | NS |
| Haemochromatosis | 1 | 0 | NS |
| Thorotrast | 1 | 0 | NS |
| Anabolic steroids | <1 | 0 | NS |
| Tyrosinaemia | <1 | 0 | NS |
| Colitis ulcerosa | <1 | 0 | NS |

[a]see text. [b]More than 6 months before diagnosis.[c]Other malignant tumours treated successfully before diagnosis of liver cancer. [d]History of alcohol abuse in 50%. FLC, fibrolamellar carcinoma; PCT, porphyria cutanea tarda; NS, not significant.

**Table III** Plausible aetiology of cirrhosis in 341 cases with hepatocellular carcinoma in Gothenburg 1958–79

| *Aetiology* | n *(%)* | *Sex M/F* |
|---|---|---|
| Alcohol | 99 (29) | 19/1 |
| Hepatitis[a] | 13 (4) | 2/1 |
| Arsenic[b] | 12 (4) | 5/1 |
| PCT[c] | 5 (1) | 3/2 |
| Thorotrast[d] | 3 (1) | 2/1 |
| Haemochromatosis | 3 (1) | 3/0 |
| PBC | 2 (1) | 0/2 |
| CAH | 2 (1) | 0/2 |
| Tyrosinaemia | 1 (<1) | 1/0 |
| Cirrhosis cardiacae | 1 (<1) | 1/0 |
| Unknown[a] | 208 (61) | 2/1 |

[a]See text. [b]History of alcohol abuse ($n = 3$), alcohol and PCT ($n = 1$), history of hepatitis ($n = 2$). [c]History of alcohol abuse ($n = 2$). [d]History of alcohol abuse ($n = 1$), PCT ($n = 1$). PCT, porphyria cutanea tarda; PBC, primary biliary cirrhosis; CAH, chronic active hepatitis.

Two cases of fibrolamellar carcinoma were found and thus constituted less than 1% of all HCC cases. Neither of these two patients had cirrhosis or any other liver disease.

There was one patient each with angiosarcoma and infantile haemangioendothelioma. The first one had undergone a radiographic examination by using the contrast dye Thorotrast 33 years before admission. Histological examination revealed angiosarcoma in a non-cirrhotic liver and deposition of Thorotrast in tumour tissue and lymph nodes. The patient with infantile haemangioendothelioma reported as PLC died post-operatively a couple of weeks after onset of symptoms.

### Discussion

The design of our study was aimed at minimising many sources of error that afflict most reports on incidence and aetiology of PLC and relative proportions of different histological types. Many reports derive from referral centres without a defined population, and thus the selection procedure may influence the results. In dealing with epidemiological data it is also important to include all known cases and that the diagnosis is correct. As is seen in the present study, re-evaluation of preserved biopsy specimens revealed diagnosis other than the reported PLC in 9%.

Our report concerns a specified region with a well-defined population, and during the study period all known cases with PLC were taken into consideration (Kaczynski and Wallerstedt, 1988). As a result of a high autopsy frequency during the study period, histologically proven diagnosis in the vast majority of cases and preservation of biopsy material for re-evaluation we had a unique opportunity to present reliable figures about the incidence and proportion of various types of PLC in a low-endemic area.

### *Incidence of HCC*

According to Anthony (1987) the relative frequency of HCC in the world expressed as number of cases per 100 000 population per year is 20–150 in high-incidence areas, 5–20 in intermediate- and less than 5 in low-incidence areas, e.g. north Europe. Our presented incidence rates confirm that Gothenburg is a low-incidence area of HCC.

### *Relative proportion of PLC subtypes*

The relative proportions of rare PLC subtypes in our study from a low-incidence area are similar to the results from a large survey from Japan (Liver Cancer Study Group of Japan, 1990), although in that study only 37% of all reported cases with PLC were histologically proven and analysed. The ratio HCC/CCC in that study was 17:1 compared with 11:1 in our study.

The main disadvantage of a retrospective, compared with a prospective study, is systematic bias. On the other hand, since the diagnosis of both HCC and CCC was known or suspected ante mortem in only about 30% of cases in our study, a prospective study would concern at most a third of the patients. Furthermore, as a result of a very low incidence (of CCC especially) in our area, such a study would be difficult to carry out. Since our report considers all known cases with PLC during the study period it was possible to compare the proportion of various aetiological factors in HCC and CCC cases.

### *HCC*

Our findings of a high frequency of liver cirrhosis in cases with HCC and, compared with populations with a high PLC incidence, a higher age of patients and a less striking male preponderance are in accordance with other reports (Lefkowitch, 1981; Anthony, 1987; Colombo, 1992). In patients with HCC and underlying liver cirrhosis of known aetiology, alcohol was by far the most important factor (Table III).

In some cases there was a history of hepatitis more than 10 years before diagnosis of HCC (Table III), but lack of preserved sera made it impossible to state the role of a previous viral hepatitis. However, as reported before (Kaczynski *et al.*, 1991), HBV seems to be of minor importance in the aetiology of HCC in our study area. The role of hepatitis C virus (HCV) in the aetiology of HCC (Simonetti *et al.*, 1991; Resnick and Koff, 1993) in our area may, however, only be answered in a prospective study.

Since liver cirrhosis is associated with risk of HCC it has been suggested that those patients should be followed regularly and screened by ultrasonography and undergo measurement of serum alpha fetoprotein (Oka *et al.*, 1994). As shown in our study, however, it must be stated that an underlying liver cirrhosis was diagnosed only in the minority of patients with HCC before development of the tumour.

History of transfusion has been correlated with risk of HCC in some studies, in at least some cases probably due to HBV and/or HCV infection (Fukuda *et al.*, 1989). We were not able to confirm this suggestion since transfusions were reported unusual in both HCC and CCC patients in our study. Diabetes has also been reported to be correlated with HCC (Lawson *et al.*, 1986; Yu *et al.*, 1991). In our study there was no significant difference in frequency of diabetes between HCC and CCC, and the prevalence of diabetes was as expected in this age group (Scherstén, 1992). Parity as a risk factor is controversial (Hsing *et al.*, 1992; Lambe *et al.*, 1993). We found a significant higher history of parity among women with HCC than among women with CCC. There was no significant difference regarding number of births between those two groups, a factor which has also been suggested as a risk factor for development of HCC (Hsing *et al.*, 1992).

Chronic exposure to inorganic arsenic has been implicated in the pathogenesis of angiosarcoma in German vineyard workers (Popper *et al.*, 1978) and after treatment with Fowler's solution (Lander *et al.*, 1975). Since inorganic arsenic may predispose for liver fibrosis (Anthony, 1987) and cirrhosis (Lander *et al.*, 1975; Popper *et al.*, 1978) it may also increase the risk of HCC. Most of our patients with HCC who had been treated with arsenic had liver cirrhosis. Development of HCC after treatment of syphilis with arsenic has not been reported previously.

Thus our data give some support to the theory that alcohol may be one of the main factors for the development of HCC in the West (Nørredam, 1979; Tamburro and Lee, 1981; Hardell *et al.*, 1984; Bassendine, 1986; Yu *et al.*, 1991).

### *CCC*

The next commonest PLC type, CCC, is reported to be a disease of older individuals, to affect both sexes equally (Okuda *et al.*, 1977; Wight, 1982; Anthony, 1987) and to account for between 5% and 30% of all cases of PLC (Okuda *et al.*, 1977; Nørredam, 1979; Bassendine, 1986;

Johnson, 1987; Altaee et al., 1991). In our study, this type was twice as common in women than in men (Table I), and although the patients were mostly old there was no significant age difference between CCC and HCC patients.

Contrary to HCC, there seems to be no association between cirrhosis and CCC. Cirrhosis in cases with CCC is sometimes believed to be a consequence of the tumour (Wight, 1982) and is reported in between 0% and 15% of all cases with CCC (Okuda et al., 1977; Johnson, 1987; Altaee et al., 1991). Almost one-third of the cases with CCC in our study had liver cirrhosis and though the frequency was significantly lower than in cases with HCC, cirrhosis may not be excluded as a contributing factor in the aetiology of CCC.

There was a remarkable correlation between cholelithiasis and CCC in our study. Cholecystectomy has been reported to decrease the risk of extrahepatic bile duct cancer and increase the risk of PLC, though only during the first year after operation (Ekbom et al., 1993). The role of gallstones in aetiology of CCC has been controversial. Although a correlation between intrahepatic gallstones and extrahepatic bile duct cancer has been found in Thailand (Chen et al., 1989), a finding of gallstones is reported in only 3-18% in most studies of CCC (Altaee et al., 1991; Okuda et al., 1977). The incidence of gallstones is reported to increase with age and may be as high as 30% in patients older than 70 years (Karran et al., 1985). Cirrhosis has also been reported to increase the risk of gallstones (Bouchier, 1969). The proportion of liver cirrhosis in patients with HCC was higher than in patients with CCC in our study, and there was no significant age difference between those two groups. Despite that, the proportion of cholelithiasis in patients with CCC was significantly higher than in patients with HCC (Table II). Thus, cholelithiasis may be an important aetiological factor in CCC in a low-incidence area of PLC.

We conclude that (a) HCC and CCC in our study population are diseases of older people with no significant difference in age between them; (b) alcohol may be one of the main factors in the development of HCC in the West; (c) liver cirrhosis is clearly correlated with the risk of HCC but is in majority of the cases unknown before diagnosis of the tumour; (d) cholelithiasis may be one of the main aetiological factors in development of CCC in a low-incidence area.

**Acknowledgements**

Grant support for this study was received from the Research Foundation against Cancer, Jubileumskliniken, Sahlgrenska Hospital, Göteborg, Sweden.

### References

ALTAEE MY, JOHNSON PJ, FARRANT JM AND WILLIAMS R. (1991). Etiologic and clinical characteristics of peripheral and hilar cholangiocarcinoma. *Cancer*, **69**, 2051-2055.

ANTHONY PP. (1987). Tumours and tumour-like lesions of the liver and biliary tract. In *Pathology of the Liver*, 2nd edn, MacSween RNM, Anthony PP and Scheuer PJ (eds) pp. 574-645. Churchill Livingstone: Edinburgh.

BASSENDINE MF. (1986). Alcohol - a major risk factor for hepatocellular carcinoma? *J. Hepatol.*, **2**, 513-519.

BASSENDINE MF. (1987). Aetiological factors in hepatocellular cancer. *Baillieres Clin. Gastroenterol.*, **1**, 1-16.

BEASLEY RP. (1988). Hepatitis B virus. The major etiology of hepatocellular carcinoma. *Cancer*, **61**, 1942-1956.

BENGMARK S, BÖRJESSON B AND HAFSTRÖM L. (1971). The natural history of primary carcinoma of the liver. *Scand. J. Gastroenterol*, **6**, 351-355.

BERMAN MM, LIBBEY NP AND FOSTER JH. (1980). Hepatocellular carcinoma. Polygonal cell type with fibrous stroma: an atypical variant with a favorable prognosis. *Cancer*, **46**, 372-379.

BOUCHIER IAD. (1969). Postmortem study of the frequency of gallstones in patients with cirrhosis of the liver. *Gut*, **10**, 705-710.

CANCER REGISTRY. (1960-82). *Cancer Incidence in Sweden 1958-1979*. National Board of Health and Welfare: Stockholm.

CHEN M-F, JAN Y-Y, WANG C-S, JENG L-BB, HWANG T-L AND CHEN S-C. (1989). Intrahepatic stones associated with cholangiocarcinoma. *Am. J. Gastroenterol*, **84**, 391-395.

COLOMBO M. (1992). Hepatocellular carcinoma. *J. Hepatol.*, **15**, 225-236.

COOK GC. (1985). Hepatocellular carcinoma: one of the world's most common malignancies (editorial). *Q.J. Med.*, **233**, 705-708.

CRAIG JR, PETERS RL, EDMONDSON HA AND OMATA M. (1980). Fibrolamellar carcinoma of the liver: a tumor of adolescents and young adults with distinctive clinico-pathologic features. *Cancer*, **46**, 2-9.

DUSHEIKO G. (1987). Hepatocellular carcinoma: molecular biology, etiology and animal models. *Gastroenterol. Clin. N. Am.*, **16**, 575-590.

EKBOM A, HSIEH C-C, YUEN J, TRICHOPOULOS D, McLAUGHLIN JK, LAN S-J AND ADAMI H-O. (1993). Risk of extrahepatic bileduct cancer after cholecystectomy. *Lancet*, **342**, 1262-1265.

FUKUDA A, SUGIMACHI K, TOKUDOME S, IKEDA M, KOGA S AND HIROHATA T. (1989). Blood transfusion as a risk factor for cirrhosis and liver cancer: a matched case - control study. *J. Natl Cancer. Inst.*, **81**, 1189-1190.

HAFSTRÖM L. (1986). Primary liver cancer in Sweden. *Ann. Chir. Gynaecol.*, **200**, (Suppl.) 11-12.

HARDELL L, BENGTSSON NO, JONSSON U, ERIKSSON S AND LARSSON LG. (1984). Aetiological aspects on primary liver cancer with special regard to alcohol, organic solvents, and acute intermittent porphyria - an epidemiological investigation. *Br. J. Cancer*, **50**, 389-397.

HEY A AND ROCKELEIN G. (1985). Primary carcinoma of the liver in autopsy material. *Fortschr. Med.*, **103**, 238-242.

HSING AW, McLAUGHLIN JK, HOOVER RN, CO CHIEN HT, BLOT WJ AND FRAUMENI JF. (1992). Parity and primary liver cancer among young women. *J. Natl Cancer Inst.*, **84**, 1118-1119.

JOHNSON PJ. (1987). The clinical features and natural history of malignant liver tumours. *Baillieres Clin. Gastroenterol.*, **1**, 17-34.

KACZYNSKI J AND WALLERSTEDT S. (1988). Registration of liver cancer data - a study on the reliability of the Swedish Cancer Registry. *Acta Oncol.*, **28**, 716-717.

KACZYNSKI J, HANSSON G, NORKRANS G AND WALLERSTEDT S. (1991). Lack of correlation between hepatitis B virus infection and the increasing incidence of primary liver cancer in Sweden. *Acta Oncol.*, **30**, 811-813.

KARRAN S, LANE RHS, TOWNEND I AND DE LA HUNT M. (1985). Calculous disease and cholecystitis. In *Liver and Biliary Disease*, 2nd edn., Wright R, Millward Sadler GH, Alberti KGMM and Karran S. (eds). pp. 1433-1462. Baillière Tindall: London.

LAMBE M, TRICHOPOULOS D, HSIEH C-C, EKBOM A AND PAVIA M. (1993). Parity and hepatocellular carcinoma. A population - based study in Sweden. *Int. J. Cancer*, **55**, 745-747.

LANDER JJ, STANLEY RJ, SUMNER HW, BOSWELL DC AND AACH RD. (1975). Angiosarcoma of the liver associated with Fowler's solution (potassium arsenite). *Gastroenterology*, **68**, 1582-1586.

LAWSON DH, GRAY JMB, McKILLOP C, CLARKE J, LEE FD AND PATRICK RS. (1986). Diabetes mellitus and primary hepatocellular carcinoma. *Q. J. Med.*, **61**, 945-955.

LEFKOWITCH JH. (1981). The epidemiology and morphology of primary malignant liver tumours. *Surg. Clin. N. Am.*, **61**, 169-180.

LIVER CANCER STUDY GROUP OF JAPAN. (1990). Primary liver cancer in Japan. *Ann. Surg.*, **211**, 277-287.

NØRREDAM K. (1979). Primary carcinoma of the liver. *Acta Pathol. Microbiol. Scand. (A)*, **87**, 227-236.

OKA H, TAMORI A, KUROKI T, KOBAYASHI K AND YAMAMOTO S. (1994). Prospective study of α-fetoprotein in cirrhotic patients monitored for development of hepatocellular carcinoma. *Hepatology*, **19**, 61-66.

OKUDA K, KUBO Y, OKAZAKI N, ARISHIMA T, HASHIMOTO M, JINNOUCHI S, SAWA Y, SHIMOKAWA Y, NAKAJIMA Y, NOGUCHI T, NAKANO M, KOJIRO M AND NAKASHIMA T. (1977). Clinical aspects of intrahepatic bile duct carcinoma including hilar carcinoma. *Cancer*, **39**, 232-246.

OKUDA K, OBATA H, NAKAJIMA Y, OHTSUKI T, OKAZAKI N AND OHNISHI K. (1984). Prognosis of primary hepatocellular carcinoma. *Hepatology*, **4**, 3S-6S.

POLTERAUER P AND ULRICH W. (1982). Primary liver cancer; results of 268 autopsies. *Onkologie*, **5**, 76-78.

POPPER H, THOMAS LB, TELLES NC, FALK H AND SELIKOFF IJ. (1978). Development of hepatic angiosarcoma in man induced by vinyl chloride, Thorotrast, and arsenic. *Am. J. Pathol.*, **92,** 349–376.

RESNICK RH AND KOFF R. (1993). Hepatitis C – related hepatocellular carcinoma. *Arch. Intern. Med.*, **153,** 1672–1677.

SCHERSTÉN B. (1992). Epidemiologi vid typ 2 - diabetes (in swedish). In *Diabetes*, Agardh, C-D, Berne C and Östman J. (eds). pp. 65–74. Almqvist & Wiksell Förlag AB.

SEEFF LB, BEEBE GW, HOOFNAGLE JH, NORMAN JE, BUSKELL-BALES Z, WAGGONER JG, KAPLOWITZ N, KOFF RS, PETRINI JL, SCHIFF ER, SHOREY J AND STANLEY MM. (1987). A serological follow-up of the 1942 epidemic of post-vaccination hepatitis in the United States army. *N. Engl. J. Med.*, **316,** 965–970.

SIMONETTI RG. CAMMA C, FIORELLO F, POLITI F, D'AMICO G AND PAGLIARO L. (1991). Hepatocellular carcinoma. A worldwide problem and the major risk factors. *Dig. Dis. Sci.*, **36,** 962–972.

TAMBURRO CH AND LEE H-M. (1981). Primary hepatic cancer in alcoholics. *Clin. Gastroenterol.*, **10,** 457–477.

VITALE GC, HEUSER LS AND POLK HC. (1986). Malignant tumors of the liver. *Surg. Clin. N. Am.*, **66,** 723–741.

WATERHOUSE J, MUIR CS, SHANMUGARATMAN K AND POWELL J. (1982). *Cancer Incidence in Five Continents*, Vol IV. International Agency for Research on Cancer: Lyon.

WIGHT DGD. (1982). Secondary carcinoma, hepatocellular carcinoma and other primary liver tumours. In *Atlas of Liver Pathology*, Wight DGD. (ed.) pp. 167–181. MTP Lancaster: MTP Press.

YU MC, TONG MJ, GOVINDARAJAN S AND HENDERSON BE. (1991). Nonviral risk factors for hepatocellular carcinoma in a low-risk population, the non-Asians of Los Angeles county, California. *J. Natl Cancer Inst.*, **83,** 1820–1826.

ZAMAN SN, JOHNSON RD, JOHNSON PJ, MELIA WM, PORTMANN BC AND WILLIAMS R. (1985). Risk factors in development of hepatocellular carcinoma in cirrhosis: prospective study of 613 patients. *Lancet*, **1,** 1357–1360.